# Qualitative attributes of meat from Teramana goat kids, an Italian native breed of the Abruzzo region

**DOI:** 10.5713/ab.21.0352

**Published:** 2022-01-03

**Authors:** Andrea Ianni, Francesca Bennato, Camillo Martino, Alessio Di Luca, Giuseppe Martino

**Affiliations:** 1 Faculty of Bioscience and Technology for Food, Agriculture and Environment, University of Teramo, Teramo (TE) 64100, Italy; 2 Istituto Zooprofilattico Sperimentale dell’Abruzzo e del Molise “G. Caporale”, Via Campo Boario, 64100 Teramo (TE), Italy

**Keywords:** Goat Meat, Lipid Oxidation, Rumenic Acid, Teramana Goat, Volatile Compounds

## Abstract

**Objective:**

The aim of this work was the characterization of the qualitative aspects of meat obtained from Teramana goats, an Italian indigenous breed of the Abruzzo region. Specifically, the study included a comparison with meat samples deriving from Saanen goat kids reared in the same environment and applying the same feeding protocol.

**Methods:**

Upon reaching about 7 months of age the animals were slaughtered and samples of muscle tissue were collected to be analyzed. Specifically, meat samples were subjected to evaluations of the physical parameters, including color and the meat ability to retain water, in addition to chemical evaluations that were focused to the determination of the total lipids amount, fatty acids composition, lipid oxidation, and volatile profile.

**Results:**

The meat samples obtained from the indigenous breed showed a less intense reddish color and no significant variations for the muscle tissue tendency to retain water, both regarding fresh and cooked meat. Several differences were instead observed in the fatty acid profile. The Teramana samples were richer in saturated fatty acids (p<0.01) and interestingly showed higher concentrations of rumenic acid (p<0.05), a conjugate of linoleic acid that has been associated with important health benefits for the consumers. Another important finding for these meat samples was the marked resistance to oxidative events, as evidenced by the thiobarbituric acid reactive substances-test (p<0.05) and by the characterization of the volatile profile that highlighted a strong reduction in the relative percentage of hexanal (p<0.05), commonly associated to lipid oxidation and the development of unpleasant aromatic notes.

**Conclusion:**

The collected data, therefore appeared useful for the valorization of the food product derived from the Teramana goat, although no sensory information has been collected to define the degree of acceptability by the consumers.

## INTRODUCTION

The evolution of social and economic conditions in the second half of the twentieth century, led to a marked increase in the demand of products of animal origin. For this reason, the livestock sector has undergone a profound change, which led to the development and spread of highly productive animal breeds, with the consequent decrease of the genetic variability previously guaranteed by the presence of local races and breeds [1]. However, in the last twenty years various strategies aimed at the preservation and recovery of biodiversity have been promulgated, and this also includes all the actions implemented to save from the risk of extinction many breeds of zootechnical interest [2].

The genetic erosion also characterized the ruminant sector and, with specific regard to goat, the extent of this phenomenon is not clear or well characterized since there is no thorough knowledge of the global population size. This aspect is mainly justified by the fact that goats are very often reared in extensive production systems, and this involves an objective difficulty for the purposes of the animal census [3]. Goats are widely distributed around the world and have been a source of human nutrition since the beginnings of human civilization [1]. In the western world, the perception of consumers is not in favour of goat meat [2], since there is a slight preference for beef, presumably due to residual effects of habit and texture preferences. However, goat meat products are without a doubt one of the staple red meats of satisfactory eating quality in several areas of the world [4,5].

In industrialized countries, there is a high distribution of cosmopolitan breeds characterized by high production indices, while the maintenance of native breeds is more associated with small and sporadic realities in which there is an attempt to apply sustainable farming systems. Such approaches are mainly distributed in marginal and disadvantaged rural areas and carry out a fundamental activity in favor of product diversification, biodiversity maintenance and preservation of landscape integrity [6]. The European territory has registered the largest portion of goat genetic resources (accounting for about the 33%), even though in Europe there is only 4% of the global goat population; an aspect that has been partially justified by the fact that Europeans were pioneers in the implementation of strategies useful for the characterization of genetic resources of zootechnical interest [3]. Among the European countries, Italy counts the largest number of local goat breeds [7] and, of particular interest for our study is the goat of Teramo (or Teramana), which is present in a few hundred specimens in the Abruzzo region and takes its name from the province of Teramo, where these animals have their greatest concentration. Only since the early 2000s has a population register been established for this goat breed, in order to carry out a detailed census and identify useful strategies for the preservation of this genetic resource at risk of extinction. These are medium-sized goats with a dark coat (mainly black or dark brown) with the possibility of white streaks on the head; the head is long with a straight frontal-nasal profile with the possibility of having horns in both sexes. Overall, the phenotypic characteristics are particularly heterogeneous due to the frequent crosses that over time occurred with goat breeds native of the southern Italy.

Only a limited number of farmers are dedicated to the breeding of these animals, and their effort in preserving these goats is more associated with the preservation of popular traditions rather than commercial purposes. Currently there is no detailed characterization of the chemical-nutritional properties associated with the meat of the Teramana goat, therefore an action of this type appears necessary to fill a lacuna of knowledge. The present study is therefore placed in a perspective of this type, assuming that it is possible to put in evidence some qualitative aspects of the Teramana goat meat, that can give greater value to this product in comparison with what is present in the reference market. Specifically, a comparison was made with meat deriving from Saanen goats which have a widespread distribution on the reference territory for both milk and meat. Therefore, Saanen goats were used as control group for evaluations that have been aimed at the characterization of meat physical properties and, above all, at the evaluation of the fatty acids (FA) profile, the lipid oxidation, as well as the accumulation of volatile compounds (VOC) in cooked meat, which give useful information for a better understanding of the oxidation phenomena and for the identification of aromatic notes that could influence the sensory profile.

## MATERIALS AND METHODS

This study was performed on a commercial farm located in the province of Teramo (Abruzzo, Italy) which usually deals with the breeding of both Saanen and Teramana goats. Animals were handled in accordance with the national legislation on animal welfare (Council Directive 2008/119/EC) [8] and slaughtered in compliance with the European Council Regulation 1099/2009 [9] which deals with the protection of animals at the time of killing. For the scope of the study, animals did not undergo breeding practices other than those commonly adopted, for this reason no further ethical statements are considered necessary.

### Experimental design, animals and feeding

In this study were used twenty male goat kids, 10 of Saanen breed (SB) and 10 of Teramana breed (TB). Only for the first 4 to 5 days of life, goat kids were kept constantly under their mothers to allow for colostral milk intake. Weaning took place at about 60 days of life, after which Saanen and Teramana goat kids were selected to be homogeneous for age. Until the time of slaughter, the animals shared an area of free housing equipped with bunks on straw, a drinking trough, and an access to the feeding area. Regarding the administered diet, every day each animal received alfalfa hay *ad libitum* with 1 kg/head/d of a concentrate whose composition is reported in Table 1. During the trial, goat kids were kept in a shared area in which were present provisional single bunks on straw, useful for the individual administration of the diet.

Feed samples were collected and analyzed for dry matter (method 930.15), ether extract (method 920.39), crude protein (method 954.01), crude fiber (method 962.09), and ash (method 942.05) according to the official methods of analysis of AOAC International [10]. The detergent procedures described by Goering and Van Soest [11] were instead applied for the determination of neutral detergent fiber, acid detergent fiber, and acid detergent lignin. Table 1 reports the FA profile of the administered diet, that was evaluated by using the same procedure described below for meat samples.

Upon reaching about 7 months of age, the goat kids were led to a commercial slaughterhouse. Each carcass was split in half covered with a film during storage to avoid contact with the surrounding environment and preserved at a controlled temperature of 4°C, following the protocol normally provided in the meat production chain.

For each animal, samples of muscle tissue were taken from the *longissimus dorsi* muscle (between the 12th and 13th ribs from the left side of the carcasses), and the sampling was performed after 3 days postmortem. During the sampling, particular attention was paid to removing only the 7 to 8 mm of the most exposed muscle tissue to standardize individual sampling.

The measurements of meat pH were directly performed at the slaughterhouse. The evaluations described below concerning drip loss, cooking loss, meat color, lipid content and FA profile were carried out only on raw meat samples collected after 3 days from slaughter. The lipid oxidation and the volatile profile were evaluated on cooked meat, both immediately after cooking (T0) and after 3 days (T3) of storage at 4°C.

Meat samples, both raw and cooked, not immediately analyzed were packed under vacuum and frozen at −20°C.

### Evaluation of pH, color, moisture, and fat content

The pH of muscle tissue was evaluated with a portable pH meter equipped with an electrode (Crison, Barcelona, Spain) that was inserted approximately 1 cm into the loin at the 12/13th rib site. Measurements were performed in triplicate after 45 min (pH_45_) and 24 hours (pH_24_) from slaughtering. Before analysis the pH meter was calibrated using standard phosphate buffers (pH 4.01 and 7.00) and each measurement was automatically adjusted for muscle temperature.

Color measurements were performed on meat samples following a blooming time of 30 min, and evaluations referenced to the chromatic coordinates of the CIELAB system that considers lightness (L*), redness (a*) and yellowness (b*). Data were obtained by using a Minolta-CR 300 (Minolta Co, Osaka, Japan) from a transverse section of the whole cut of the muscle tissue. The instrument was characterized by a D65 illuminant, an aperture size of 32 mm and 10 standard observer angle. The instrument calibration was prosecuted by exploiting a white tile (L* = 100) and a black glass (L* = 0).

The evaluations of moisture and fat content were performed on preventively minced raw meat by following the procedures reported by Horwitz and Latimer [12].

### Drip loss and cooking loss

The drip loss was assessed by preparing meat sections of approximately 2.0 cm thickness and 50 to 60 grams in weight. The meat samples were then inserted in an expanded bag and left at 4°C; after 48 hours the meat samples surface was slightly blotted with an absorbent paper tissue and reweighed. The drip loss was then expressed as a percentage with respect to the initial sample weight.

Goat meat samples of 70 to 75 g of weight and about 2.0 cm of thickness were cooked in a water bath (Grant Instruments Ltd., Barrington, UK) until reaching a core temperature of 75°C (Minitherm HI8751 temperature meter and probe; Hanna Instruments Ltd., Bedfordshire, UK). Cooked samples were then tempered at 18°C to 20°C (room temperature) and left overnight to cool at 4°C. The cooking loss values were then expressed as a percentage of the starting sample weight.

### Fatty acid profile and lipid oxidation

The total lipids extraction was obtained through the Folch method [13]. About 2.5 g of preventively minced meat were homogenized in UltraTurrax T-25 in 40 mL of a solution composed by chloroform and methanol (2:1, v/v). Samples were then left in continuous stirring for 6 hours at room temperature and filtered overnight in a separating funnel in presence of sodium chloride. The chloroform phase containing the lipid fraction was recovered and then evaporated to dryness at 40°C with a Strike-Rotating Evaporator (Steroglass S.r.l., Perugia, Italy).

For each sample, 60 mg of the extracted fat were weighed and mixed with 6 mL of hexane. The formation of the fatty acid methyl esters (FAME) was achieved by adding in the solution 100 μL of methanol and 100 μL of sodium methoxide. The identification of individual FAMEs was performed by using a gas chromatograph with a flame ionization detector (GC-FID; Thermo Scientific, Waltham, MA, USA) equipped with a capillary column (Restek Rt-2560; Restek Corporation, Bellefonte, PA, USA) and exploiting hydrogen as carrier gas. The ChromeCard software was used for the quantification of peak areas, and the values associated to individual FAs were expressed as relative percentages of total FAMEs. To identify individual FAMEs, a comparison was made with the retention time of the standard mixture FIM-FAME7-Mix (Matreya LLC, State College, PA, USA). The percentage value that was attributed to each FA was helpful in calculating the sum of saturated fatty acids (SFA), monounsaturated fatty acids (MUFA), and polyunsaturated fatty acid (PUFA). Desaturation indices were instead calculated by using the formula suggested by Brogna et al [14].

The lipid oxidation was evaluated by the thiobarbituric acid reactive substances-test (TBARS-test) method following the protocol previously applied by Ianni et al [15].

### Volatile profile of cocked meat

Five grams of previously minced cooked meat were mixed with 10 mL of a NaCl solution (360 g/L) in which were previously added 10 μL of the internal standard solution (3-methyl-2-heptanone; stock solution of 10 mg/L in ethanol). Volatile compounds were extracted from the headspace with a divinylbenzene-carboxen-polydimethylsiloxane solid-phase microextraction fiber (length, 1 cm; film thickness, 50/30 m; Sigma-Aldrich, Milan, Italy) with an exposition time of 45 min at 50°C. The extracted VOCs were then thermally desorbed with a splitless mode into the GC (Clarus 580; Perkin Elmer, Waltham, MA, USA) equipped with an Elite-5MS column (length internal diameter, 30×0.25 mm; film thickness, 0.25 m; Perkin Elmer, USA) and coupled with a mass spectrometer (SQ8S; Perkin Elmer, USA). The applied thermal program, as well as the identification of the individual VOCs were performed as previously reported [16].

### Statistical analysis

All the analyzes described were performed on ten replicates (n = 10) for each treatment (SB vs TB), and the obtained results were reported as mean values±standard deviation. The software SigmaPlot 12.0 (Systat software Inc., San Jose, CA, USA) for Windows operating system was used to evaluate the statistical significance of the observed variations (analysis of variance and Student’s t-test); specifically, statistical significance was attributed in presence of p values lower than 0.05.

Regarding the TBARS-test, the comparison between Teramana and Saanen goat kids was performed independently after 0 (T0) and 3 (T3) days of cooked meat storage; therefore, the two datasets were processed separately and the effect of the time of storage was not tested.

## RESULTS

### Physical and chemical properties of goat meat

The comparison performed in physical and chemical properties of goat meat between SB and TB muscle tissue samples (Table 2) did not highlight significant differences regarding pH, moisture, total lipid amount and in the ability to retain water respectively by fresh (drip loss) and cooked (cooking loss) meat samples.

Also, in the case of color, no variations were observed in the brightness (L*) and yellowness (b*) of the analyzed samples. However, TB showed a significant reduction in the a* parameter associated with redness (0.66±0.08 vs 0.31±0.05 in SB and TB samples respectively; p<0.01).

### Fatty acid profile and oxidative stability of meat

The characterization of the FA profile in goat meat (Table 3) showed TB samples to be significantly richer in palmitic (C16:0; p<0.05), stearic (C18:0; p<0.05), trans vaccenic (C18:1 trans-11; p<0.05) and rumenic (C18:2 cis-9, trans-11; p<0.05) acids, while a reduced concentration of oleic acid (C18:1 cis-9; p<0.01) was observed.

The evaluations performed on individual FAs were also useful in determining the total sum of SFA, MUFA and PUFA, finding a significantly higher concentration of SFA in TB samples (52.28%±4.91% vs 47.40%±3.89% in TB and SB samples respectively; p<0.01). No significant variations were instead identified for the remaining two families of compounds (p> 0.05).

With regard to the desaturation indices, significant variations were observed in relation to values calculated for myristoleic (C14:1 cis-9) and palmitoleic (C16:1 cis-9) acids, that were higher in SB samples (p<0.05), and for rumenic acid (C18:2 cis-9, trans-11) for which a more efficient desaturation was found in TB samples (p<0.05).

**Figure 1:**
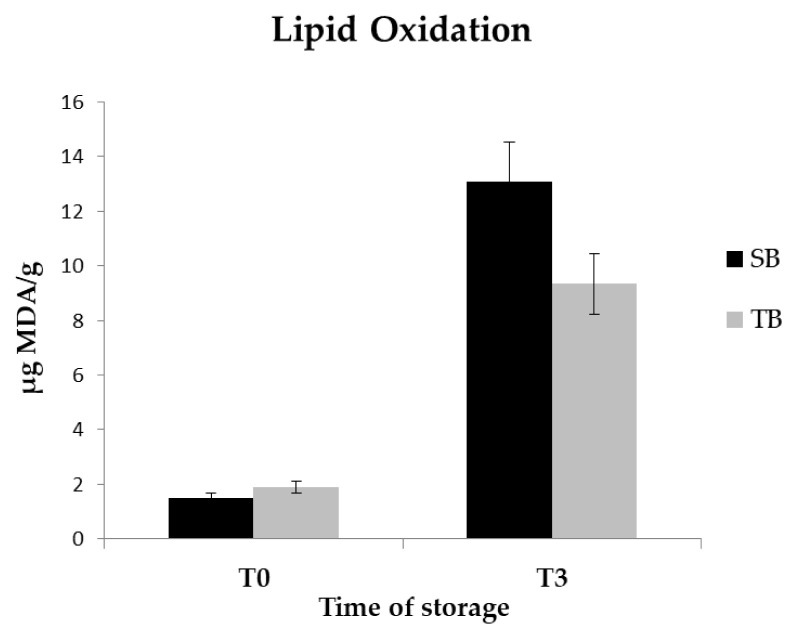
Oxidation profile detected in cooked goat meat immediately after cooking (T0) and after 3 days of storage (T3) at 4°C. Data are reported as μg of malondialdehyde (MDA) per g of sample±standard deviation. Black bars represent the values related to Saanen breed (SB), while the gray bars represent the values related to Teramana breed (TB).

The TBARS-test (Figure 1) was effective in highlighting the extent of lipid oxidation in goat meat samples subjected to heat treatment. No significant differences were observed between SB and TB samples immediately after cooking, while, after 3 days of meat storage at 4°C, the TB samples showed a better oxidative stability (13.08±1.41 μg malondialdehyde [MDA]/g vs 9.34±0.89 μg MDA/g in SB and TB samples respectively; p<0.05).

### Volatile compounds in cooked meat

The characterization of volatile profile in goat meat samples subjected to cooking was useful for the identification of 20 compounds (Table 4). Immediately after the heat treatment, the only significant variation was observed for 2-heptanal that was higher in TB samples (4.70%±0.32% vs 3.76%±0.26% in TB and SB samples respectively; p<0.05). After 3 days of cooked meat storage at 4°C, hexanal was confirmed as the most represented VOC, with a higher relative percentage in SB samples (33.17%±2.21% vs 27.37%±1.93% in TB and SB samples respectively; p<0.05). In addition to this, significantly higher concentrations of decanal (p<0.05) and 1-octen-3-ol (p<0.05) were also observed in TB samples.

## DISCUSSION

The present study was focused on the characterization of the chemical-nutritional properties of goat meat obtained from an indigenous breed of the central Italy, the Teramana goat. Specifically, the study involved only 10 Teramana goat kids, an aspect that could represent a limitation of the study. However, it must be considered that the study focuses on a goat breed at risk of extinction, so it is very difficult to have this number of animals in a single farm. Given the current condition it would be possible to have higher numbers only by combining animals from different farms, but in that case additional variables related to the environment, diet, etc. would be introduced. In any case, we believe the applied experimental design was effective in highlighting interesting aspects, although the preliminary nature of this experimentation should be reiterated.

The performed comparison did not highlight significant differences regarding pH, moisture and in the ability to retain water respectively by fresh (drip loss) and cooked (cooking loss) meat samples. The obtained pH values agreed with those previously reported by other authors for different suckling kid genotypes, both with reference to the surveys carried out to the slaughter vicinity and after 24 hours of carcass preservation [17]. As regards the ability of kid muscle tissue to retain water, it is indicative the comparison with the study conducted by Ekiz et al [18], who performed evaluations on the carcass traits and meat quality characteristics of dairy suckling kids (Turkish Saanen and Maltese) compared to an indigenous genotype (Gokceada). In this case, both values respectively associated with drip and cooking loss did not show significant differences, with values in both cases fully comparable with those obtained in the present study.

In the case of meat color, no variations were observed both for lightness (L*) and yellowness (b*); however, TB showed a significant reduction in the a* parameter that is associated with redness. The meat color is highly variable parameter generally used by consumers to judge the freshness and the overall quality of the product at the moment of purchase [19]. For instance, more yellow and dark meats are usually perceived by consumers as meats deriving from older animals or animals endowed of non-optimal health conditions. With regard to brightness, this finding can be justified, at least in part, by the lack of significant differences in the moisture content, since these parameters are very often correlated in food matrices of various origins [20,21]. The lower a* value in TB samples is instead a finding already found in the comparison between cosmopolitan and indigenous breeds [18]. Specifically, the genotype has been reported as one of the main factors capable of influencing this parameter, together with production system, ultimate carcass pH, weaning status and sex [19,22]. However, in our study these last parameters are all similar between the animals considered, so we can refer this color difference only to the genetic component.

Also, regarding the fat content, no significant variations have been highlighted, a fact which is nevertheless surprising as it is known that the genetic aspect is effective in inducing variations at the level of fat deposition. Indigenous breeds have generally a greater tendency to accumulate fat both subcutaneously and intramuscularly in response to the need to store useful energy for survival in difficult climatic environments; a phenomenon which has been better characterized in monogastrics by exploiting molecular tools [23].

Several and interesting differences were instead found in the relative abundance of individual FAs. First, the TB meat samples were found to be much richer in SFA, a figure totally justified by the presence of significantly higher concentrations of palmitic (C16:0) and stearic (C18:0) acids. Since all the animals involved in this study were subjected to the same diet, the most plausible justification for these differences must certainly be sought endogenously. Taking into consideration the fact that the desaturation indices associated with palmitoleic (C16:1) and oleic (C18:1) acids are higher in SB goats, it is plausible that in the Teramana goats there is a lower expression or simply a reduced activity of the enzymes involved in these mechanisms, among all the stearoyl-CoA desaturase (SCD) [24]. This hypothesis is justified, at least in part, also by the lower presence of C18:1 and C16:1 in TB samples (for C16:1 the p value is close to significance, p = 0.063). Such an eventuality would obviously deserve to be better characterized from the molecular point of view, assuming that in other studies the high genetic variability associated to caprine SCD has already been observed. Specifically, evaluations performed on different breeds evidenced the presence of gene polymorphism; a condition that undoubtedly has direct repercussions on the kinetic parameters associated with the activity of the enzyme at the tissue level, and therefore on the FA composition of goat meat and milk [25]. In addition to what has been reported, a molecular investigation in this case should not ignore evaluations of other factors involved in the metabolism of FAs, such as the degree of expression and activity of the fatty acid synthase (FAS), a multifunctional enzyme responsible for the de novo production of palmitate starting from acetyl-coA, malonyl-CoA, and nicotinamide adenine dinucleotide phosphate [26]. It is in fact plausible that the Teramana goat is also characterized by a higher constitutive expression of this enzyme, with a significant impact on FAs biosynthesis.

The higher concentration of SFA in TB meat samples tends to be quite controversial as it is known that the consumption of foods rich in these compounds is associated with an increased risk of getting chronic diseases affecting the cardiovascular system [27]. However, from the point of view of the health potential, it should be highlighted that the Teramana goat has a higher concentration of rumenic acid (C18:2 cis-9, trans-11), which is credited of important anti-inflammatory and antioxidant functions and therefore play a role of considerable interest for the consumer [28]. Specifically, this FA accumulates in the animal tissues following desaturation events involving the vaccenic acid (C18:1 trans-11) deriving from ruminal biohydrogenation process of dietary unsaturated FAs [29]. Indeed, vaccenic acid has significantly higher concentrations in TB meat and the desaturation index associated with conjugated linoleic acid is also higher in the same samples.

In addition to what has been reported, a reduced presence of unsaturated forms is also generally associated with a lower predisposition to lipid oxidation that occurs during raw product storage or following the application of heat treatments [30]. This occurrence was firstly evaluated in our study through the analysis of the thiobarbituric acid reactive substances in cocked goat meat samples, carrying out evaluations immediately after the heat treatment and after 3 days of storage at 4°C. Such approach effectively showed a lower oxidation in TB samples at the end of the storage period, evidence that is presumably explained precisely in the reduced concentration of unsaturated FAs since a greater intake of dietary antioxidants is unlikely, due to the presence of the same feeding protocol.

The evaluation of the volatile profile in cooked meat samples had the dual purpose of highlighting any markers of lipid oxidation, and therefore of confirming what has already been observed with TBARS, and, at the same time, of obtaining useful information about the presence of any differences in the meat flavor. Overall, 20 compounds have been identified, including those that in the literature are considered to be the major VOCs of cooked meat, such as 1-octen-3-ol, hexanal, heptanal, octanal, and 1-hexanol [31]. The VOC present in higher concentration in all the samples at the end of the storage period is represented by the hexanal, which mainly derives from oxidation events involving linoleic and arachidonic acids [32]. The fact that this compound is less concentrated in the TB samples, represents a significant data as it is symptomatic of a better resistance of the sample to oxidative processes, furthermore there are positive re-percussions also on the sensory aspect, as the accumulation of this compound it is instead associated with the development of rancid aromatic fragrance able to negatively influence the degree of acceptability by the consumer [33]. A further noteworthy element concerns the finding related to the 1-octen-3-ol. In previous studies performed on turkey meat and beef muscles, this VOC, in the same way of the hexanal, was associated with the onset of oxidative processes and therefore of off-flavor [34], so it is quite unexpected that this compound is present in higher relative percentages in the TB samples which tested a better overall resistance to the oxidative process. In this case, it would be therefore useful to carry out further analyzes to verify the synthesis mechanism of this compound, without forgetting sensorial evaluations to verify if this change may have any effect on consumer acceptability.

In conclusion, the characterization of the qualitative attributes of meat obtained from the Teramana goat, was useful in the identification of some aspects that could be of interest for the valorization of the animal product. The comparison performed with meat deriving from SB, allowed first of all to observe a higher concentration of rumenic acid, which is credited of important anti-inflammatory and antioxidant functions, therefore playing a role of considerable interest for the consumer; furthermore the Teramana meat also showed richer in SFA, a condition that, although it is less compatible with the healthy functionality of the product, represents the most probable reason for the observed resistance to oxidative processes. This latter evidence therefore moves in the direction of an increased product safety, although the issue related to the sensory aspect and the consumer acceptability remains still open.

## CONCLUSION

The present study gathered useful information for the valorization of meat derived from the Teramana goat, a native breed of the central Italy. Specifically, the Teramana samples were richer in rumenic acid, a conjugate of linoleic acid, the presence of which in animal products is commonly associated to important health benefits for consumers. Furthermore, the same samples showed a better oxidative stability, as evidence of a greater ability of this meat to be conserved for longer times without the risk of unwanted phenomena that could alter its nutritional profile. In any case, the issue relating to the sensory profile, useful for determining the degree of acceptability of these products by consumers, remains open.

## Figures and Tables

**Table 1:** Ingredient and chemical composition of the administered diet

Chemical composition of alfalfa hay (%)	
DM	87.50
Ash^1)^	7.35
CP^1)^	17.40
EE^1)^	2.25
NDF^1)^	47.20
ADF^1)^	38.80
ADL^1)^	10.00
Ingredients of concentrate	
Soybean, meal	17.40
Wheat, bran	20.10
Barley, meal	31.90
Corn, meal	28.60
Vitamin and mineral	2.00
Chemical composition of concentrate	
DM	88.10
Ash^1)^	5.05
EE^1)^	3.20
CP^1)^	17.95
NDF^1)^	12.73
ADF^1)^	5.91
ADL^1)^	1.16
Starch^1)^	48.51
Fatty acid profile^2)^	
C14:0	0.75±0.08
C16:0	18.85±1.61
C16:1 c9	0.65±0.07
C18:0	8.85±0.25
C18:1 c9	23.80±2.07
C18:1 c11	0.90±0.08
C18:2 c9, 12	32.70±2.95
C18:3 c9, 12, 15	9.80±0.89
C20:0	0.50±0.06
Others	3.20±0.25

DM, dry matter; CP, crude protein; CP, crude protein; EE, ether extract; NDF, neutral detergent fiber; ADF, acid detergent fiber; ADL, acid detergent lignin; FAMEs, fatty acid methyl esters; SD, standard deviation.

1) DM basis.

2) Data are reported as mean relative percentages of total FAMEs±SD.

**Table 2:** Main physical and chemical properties of goat meat obtained from Saanen breed (SB) and Teramana breed (TB)

Trait	SB	TB	p-value
pH45^1)^	6.42±0.03	6.33±0.08	ns
pH24^1)^	5.88±0.12	5.82±0.06	ns
Drip loss (%)	1.93±0.24	2.08±0.37	ns
Cooking loss (%)	23.89±2.93	22.92±3.83	ns
L*^2)^	17.70±1.66	17.54±2.12	ns
a*^2)^	0.66±0.08	0.31±0.05	^**^
b*^2)^	3.03±0.53	3.11±0.49	ns
Chemical composition (%)			
Moisture	77.07±4.13	78.63±2.60	ns
Dry matter (DM)	22.97±4.13	21.27±2.60	ns
Total lipids^3)^	6.87±0.84	7.00±0.69	ns

All data are reported as mean±standard deviation (SD).

1) pH_45_, pH 45 min *post mortem*; pH_24_, pH 24 hours *post mortem*.

2) L*, lightness; a*, redness; b*, yellowness.

3) Data are reported on a dry matter (DM) basis.

ns , not significant;

** p<0.01.

**Table 3:** Fatty acid profile in goat meat obtained from Saanen breed (SB) and Teramana breed (TB)

Fatty acid^1)^	SB	TB	p-value
C14:0	3.03±0.38	3.49±0.62	ns
C15:0	0.70±0.08	0.68±0.11	ns
C16:0	22.29±1.59	24.77±1.85	^*^
C17:0	1.78±0.20	1.59±0.18	ns
C18:0	19.47±1.27	21.60±1.46	^*^
C20:0	0.13±0.02	0.15±0.03	ns
SFA	47.40±3.89	52.28±4.91	^**^
C14:1	0.21±0.03	0.20±0.03	ns
C16:1	2.11±0.13	1.97±0.10	ns
C18:1 c11	1.79±0.08	1.79±0.09	ns
C18:1 c9	34.03±2.73	28.71±2.48	^**^
C18:1 t11	1.19±0.13	1.40±0.12	^*^
MUFA	39.33±3.46	34.17±3.13	ns
C18:2 c9,12	8.01±0.76	8.39±0.98	ns
C18:2 c9, t11	0.68±0.07	0.89±0.07	^*^
C18:3 c9,12,15	1.29±0.04	1.27±0.16	ns
C20:4 c5,8,11,14	1.05±0.18	0.79±0.09	ns
PUFA	11.03±0.92	11.34±1.01	ns
Others	2.26±0.18	2.31±0.20	ns
DI C14:1 cis-9/(C14:0+C14:1 cis-9)	0.065±0.007	0.054±0.007	^*^
DI C16:1 cis-9/(C16:0+C16:1 cis-9)	0.086±0.009	0.074±0.008	^*^
DI C18:1 cis-9/(C18:0+C18:1 cis-9)	0.64±0.07	0.57±0.07	ns
DI CLA/(C18:1 trans-11+CLA)	0.27±0.03	0.38±0.04	^**^

1) Data are reported as mean percentages of total fatty acid methyl esters±standard deviation.

SFA, saturated fatty acid; MUFA, monounsaturated fatty acid; PUFA, polyunsaturated fatty acid; DI, desaturation index; CLA, conjugated linoleic acid.

* p<0.05;

** p<0.01; ns, not significant.

**Table 4:** Volatile compounds (VOC) detected in goat meat from Saanen breed (SB) and Teramana breed (TB), immediately after cooking (T0) and after 3 days of storage (T3) at 4°C

VOC^1)^	T0			T3		
	
	SB	TB	p-value	SB	TB	p-value
Pentanal	1.84±0.16	1.85±0.17	ns	0.91±0.11	0.82±0.09	ns
Hexanal	26.74±2.04	26.08±2.18	ns	33.17±2.21	27.37±1.93	^**^
Heptanal	1.04±0.02	1.02±0.01	ns	0.14±0.02	0.13±0.01	ns
2-Heptanal	3.76±0.26	4.70±0.32	^*^	4.69±0.37	4.49±0.32	ns
Octanal	4.75±0.41	5.48±0.46	ns	3.30±0.28	3.85±0.33	ns
Nonanal	13.64±1.08	12.63±1.22	ns	11.68±1.15	12.47±1.06	ns
Decanal	0.23±0.03	0.26±0.04	ns	0.19±0.03	0.28±0.04	^*^
1-Pentanol	1.92±0.21	1.89±0.19	ns	1.20±0.11	1.07±0.09	ns
1-Hexanol	1.10±0.12	1.07±0.13	ns	0.89±0.10	1.01±0.12	ns
1-Octen-3-ol	11.44±1.02	12.40±1.16	ns	7.15±0.66	10.87±0.98	^**^
2-Octen-1-ol	2.06±0.21	2.13±0.25	ns	3.54±0.33	4.06±0.39	ns
Cyclohexanol, 2,4-dimethyl	0.17±0.03	0.18±0.02	ns	0.19±0.03	0.20±0.02	ns
2-Heptanone	0.11±0.01	0.11±0.02	ns	0.21±0.03	0.18±0.02	ns
2-Octenal	0.64±0.07	0.58±0.07	ns	0.34±0.04	0.40±0.03	ns
2-Decenal	0.70±0.07	0.64±0.07	ns	0.54 ± 0.06	0.66±0.07	ns
Caproic Acid, vinylester	8.69±0.81	9.27±1.03	ns	8.92 ± 1.18	10.78±0.99	ns
Ethylbenzene	11.89±0.96	10.79±1.03	ns	9.58 ± 0.88	8.47±0.87	ns
P-Xylene	7.49±0.71	7.23±0.74	ns	9.85 ± 1.07	8.94±0.78	ns
O-Xylene	1.66±0.12	1.62±0.17	ns	3.23 ± 0.38	3.61±0.31	ns
Benzaldehyde	0.14±0.02	0.17±0.03	ns	0.29 ± 0.04	0.34±0.04	ns

1) Data are reported as mean relative percentage of total volatile compounds (VOC)±standard deviation.

* p<0.05;

** p<0.01; ns, not significant.
